# Compressed Video Quality Index Based on Saliency-Aware Artifact Detection

**DOI:** 10.3390/s21196429

**Published:** 2021-09-26

**Authors:** Liqun Lin, Jing Yang, Zheng Wang, Liping Zhou, Weiling Chen, Yiwen Xu

**Affiliations:** Fujian Key Lab for Intelligent Processing and Wireless Transmission of Media Information, College of Physics and Information Engineering, Fuzhou University, Fuzhou 350002, China; lin_liqun@fzu.edu.cn (L.L.); 201127079@fzu.edu.cn (J.Y.); n191120078@fzu.edu.cn (Z.W.); n181120080@fzu.edu.cn (L.Z.); weiling.chen@fzu.edu.cn (W.C.)

**Keywords:** video quality assessment, saliency detection, perceivable encoding artifacts, Dense Convolutional Network (DenseNet)

## Abstract

Video coding technology makes the required storage and transmission bandwidth of video services decrease by reducing the bitrate of the video stream. However, the compressed video signals may involve perceivable information loss, especially when the video is overcompressed. In such cases, the viewers can observe visually annoying artifacts, namely, Perceivable Encoding Artifacts (PEAs), which degrade their perceived video quality. To monitor and measure these PEAs (including blurring, blocking, ringing and color bleeding), we propose an objective video quality metric named Saliency-Aware Artifact Measurement (SAAM) without any reference information. The SAAM metric first introduces video saliency detection to extract interested regions and further splits these regions into a finite number of image patches. For each image patch, the data-driven model is utilized to evaluate intensities of PEAs. Finally, these intensities are fused into an overall metric using Support Vector Regression (SVR). In experiment section, we compared the SAAM metric with other popular video quality metrics on four publicly available databases: LIVE, CSIQ, IVP and FERIT-RTRK. The results reveal the promising quality prediction performance of the SAAM metric, which is superior to most of the popular compressed video quality evaluation models.

## 1. Introduction

Video coding technology largely reduces storage capacity and transmission bandwidth. However, lossy compression and transmission via changeable channel inevitably cause various distortions. Thus, compressed video often shows visually annoying distortions, named Perceivable Encoding Artifacts (PEAs), which greatly affect video perceived quality [1].

For effective analysis and improvement of user experience, it is necessary to accurately evaluate visual quality of video. Subjective Video Quality Assessment (VQA) is the most accurate and reliable reflection of human perception, because it is the quality scored by viewers. At present, only the results of subjective quality evaluation are used as a benchmark to measure the accuracy of objective quality evaluation methods. According to the standard given by International Telecommunications Union (ITU) [2], Mean Opinion Score (MOS) and Different Mean Opinion Score (DMOS) are employed to expressed video subjective quality. Therefore, MOS and DMOS are the most reliable quality indicators and are used to assessment objective quality of videos. However, subjective experiments are tedious, time-consuming and expensive. Consequently, it is imperative to establish reliable objective VQA index.

According to the availability of reference, the objective VQA metrics can be categorized into Full-Reference (FR), Reduced-Reference (RR) and No-Reference (NR) metrics. Typical FR metrics such as Peak Signal to Noise Ratio (PSNR) and Structural SIMilarity (SSIM) [3] have been extensively applied. RR-VQA metrics, such as Spatio-Temporal RR Entropic Differences (STRRED) [4] and Spatial Efficient Entropic Differencing for Quality Assessment (SpEED-QA) [5], also show good performance. In real-life video display, the unimpaired original video source is inaccessible to end users, thus NR metric is highly desirable. It is also the most difficult one among three types of VQA metrics due to the lack of prior knowledge from reference video. However, it is the most widely used in different applications.

As mentioned above, NR-VQA metrics have a wide range of applications, but they require that the extracted features are not sensitive to the video content and highly related to the degree of distortion. Furthermore, they have high computational complexity and still have room for improvement in the accuracy. With the development of the Natural Video Statistic (NVS) model, researchers extracted features from natural scenes. These features can describe the temporal and spatial statistical characteristics of video, and were fed into the regression model (RM) to realize the evaluation of video quality in the transform domain [6,7,8]. Motivated by the effort of unsupervised feature learning for NR image quality assessment [9], Xu et al. [10] presented a NR-VQA algorithm named video COdebook Representation for No-reference Image quality Assessment (CORNIA), where a linear Support Vector Regression (SVR) is utilized to predict the video quality based on frame-level features. In [11], a blind NR-VQA model was developed by using the statistical properties in natural videos. The model employs the output data to directly predict the video quality, without any external information about the reference video such as subjective quality score. Zhu et al. [12] presented a blind VQA method considering the characteristics of human visual system (HVS). Reddy et al. [13] proposed a NR-VQA metric utilizing an asymmetric generalized gaussian distribution model, which performs the statistics of the characteristic parameters in natural videos.

The distortion-specific NR-VQA approaches assess video quality under the premise that distortion types of video are known. In [14], a method was proposed to measure the perceived strength of blocking artifact in decoded video at the position of the non-fixed grid. Next, it was combined with the entropy measurement to predict video quality. Amor et al. [15] proposed a NR-VQA index based on blocking artifact estimation in spatial domain by calculating the difference of gray-level conversion between adjacent blocks. Xue et al. [16] proposed a VQA metric method to evaluate the impact of frame freezing caused by packet loss or delay on perceived quality. In [17], a NR-VQA model based on discrete cosine transform was developed to measure distortion, such as blocking, clearness and noise, in which a multilayer neural network was used to obtain the prediction scores of videos. A model was built by Men et al. [18] to achieve the prediction of video quality scores by combining features including blurring artifact, contrast and color bleeding. In [19], blocking, packet-loss and freezing artifacts were obtained to predict video quality. Rohil et al. [20] developed a holistic NR-VQA model based on quantifying certain distortions in video frames, such as ringing and contrast distortion. Next, the intensity values of various distortions were input to the neural network to evaluate the quality of videos. In summary, most of the existing NR-VQA algorithms are aimed at traditional videos. Some algorithms involve the transmission distortion caused by channel error, such as packet loss and frame freezing. However, there are few NR-VQA researches on compressed videos, and most of existing works only detect a single type of compression artifact. These methods can not abundantly reflect the impact of PEAs on HVS. Therefore, it is necessary to develop a NR compressed video quality evaluation algorithm combined with two or more PEAs, which shows highly correlated with subjective perception quality.

To further improve the NR-VQA performance, it is feasible to detect more artifacts. In this paper, we propose a NR-VQA metric named Saliency-Aware Artifact Measurement (SAAM) to estimate video quality by analyzing four typical types of spatial PEAs including blocking, blurring, color bleeding and ringing. We also exploit visual saliency detection and patch segmentation of interested regions to map the PEA intensities to objective score with reduced complexity. We sum up major contributions of this work as follows.

(1) Proposed a NR-VQA method based on PEA detection. The PEA detection module accurately identifies four typical types of PEAs (i.e., blurring, blocking, ringing and color bleeding). Based on the PEA detection module, the PEA intensities are obtained to analyze video quality.
(2) Introduced visual saliency detection and patch segmentation for high VQA accuracy and reduced complexity. The visual saliency detection can make useful information of videos and maximize utilization of computing resources, as well as help to eliminate the impact of redundant visual information on subjective evaluation.
(3) Achieved the superior performance of our method in terms of compressed videos. Compared with multiple typical VQA metrics, our index has the highest overall correlation coefficient with the subjective quality score. In addition, our algorithm can achieve reasonable performance in cross-database verification, which shows that our algorithm has good generalization and robustness.

## 2. PEA-Based Video Quality Index

**Figure 1:**
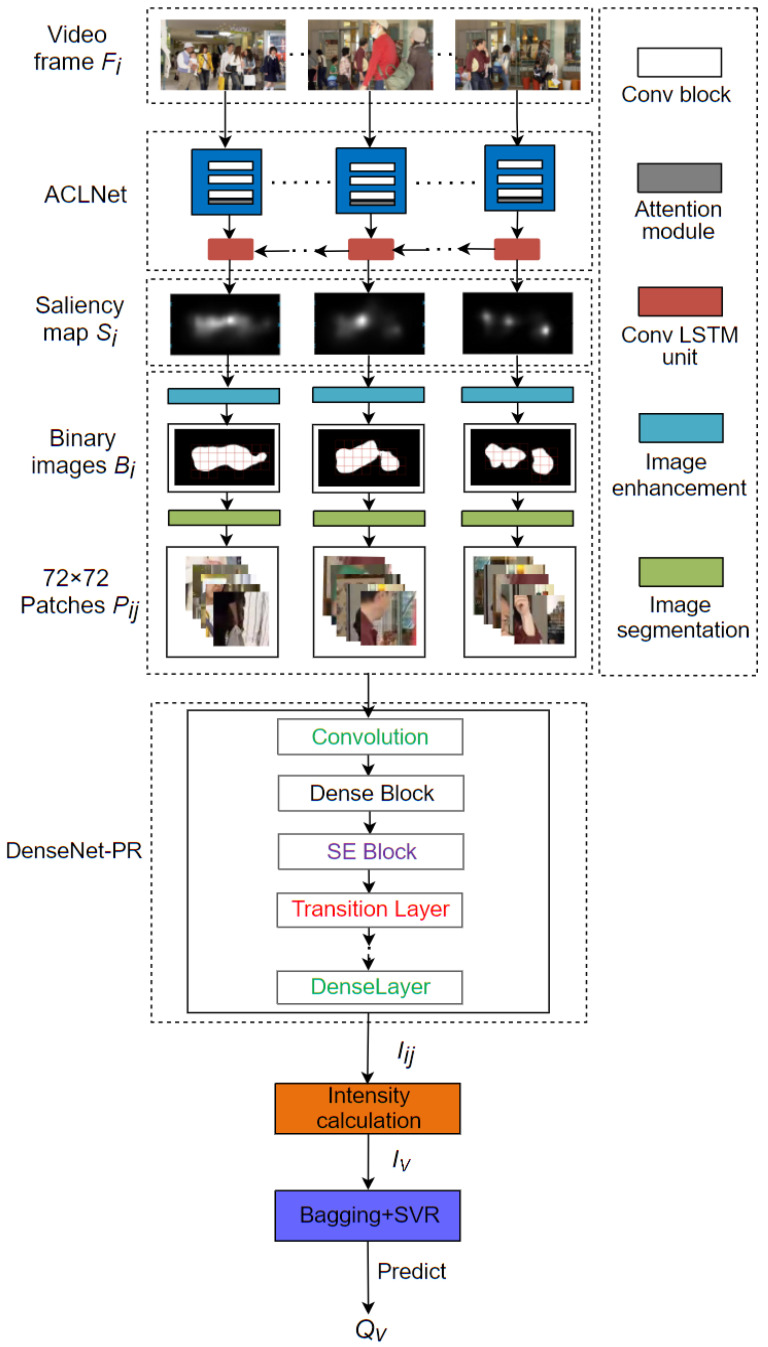
The overall architecture of SAAM.

The overall architecture of the SAAM metric is shown in Figure 1. It consists of four steps: video saliency detection with Attentive CNN-LSTM Network (ACLNet) [21] (input video frame $F_{i}$, output saliency map $S_{i}$), image patch segmentation (enter saliency map $S_{i}$ to guide the generation of 72 × 72 image patches $P_{\mathrm{ij}}$), PEA detection (input image patches $P_{\mathrm{ij}}$, output PEA intensities $I_{\mathrm{ij}}$ of patches) and SVR prediction (input PEA intensities $I_{V}$ of video, output predicted quality $Q_{V}$). These detail contents are elaborated as follows.

### 2.1. Perceivable Encoding Artifacts

In this section, we review PEA classification in [1] and select typical PEAs (including blocking, blurring, ringing and color bleeding) to develop our SAAM algorithm.

#### 2.1.1. Four Typical PEAs

The causes and manifestations of four typical types of PEAs are summarized as follows.

**Figure 2:**
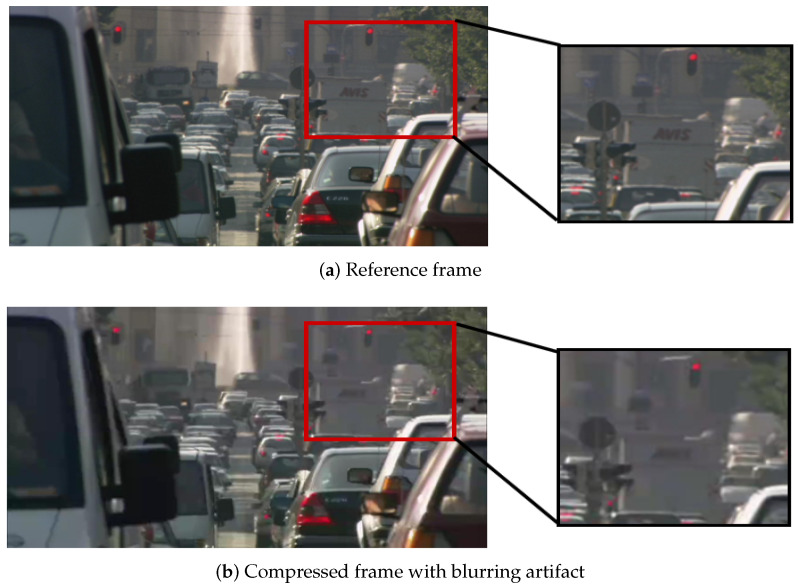
An example of blurring [23,24,25].

**Figure 3:**
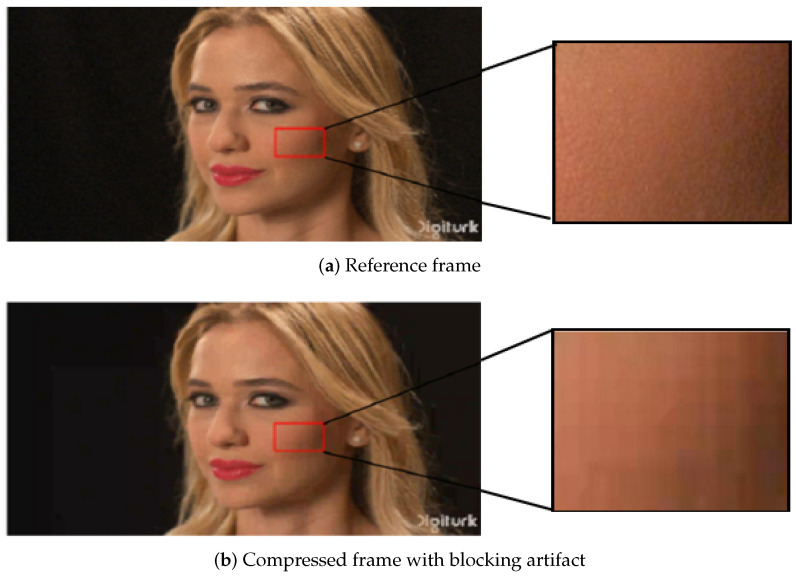
An example of blocking [27].

**Figure 4:**
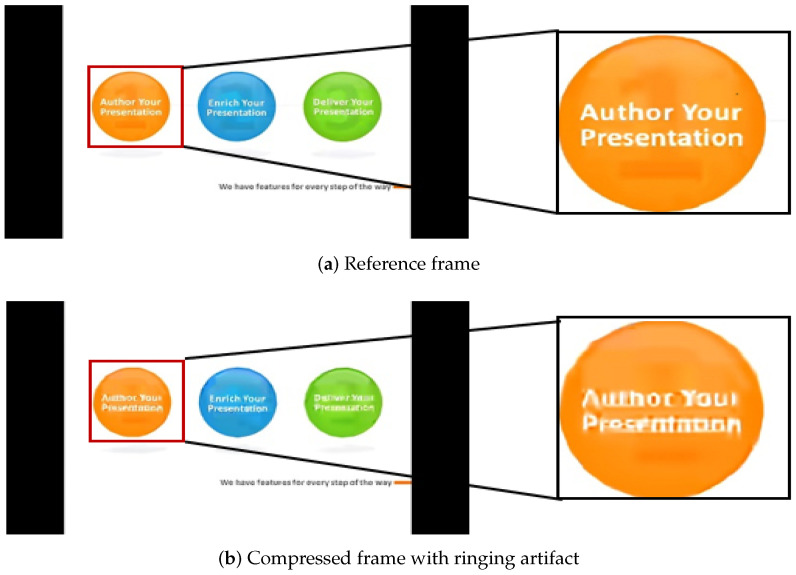
An example of ringing.

**Figure 5:**
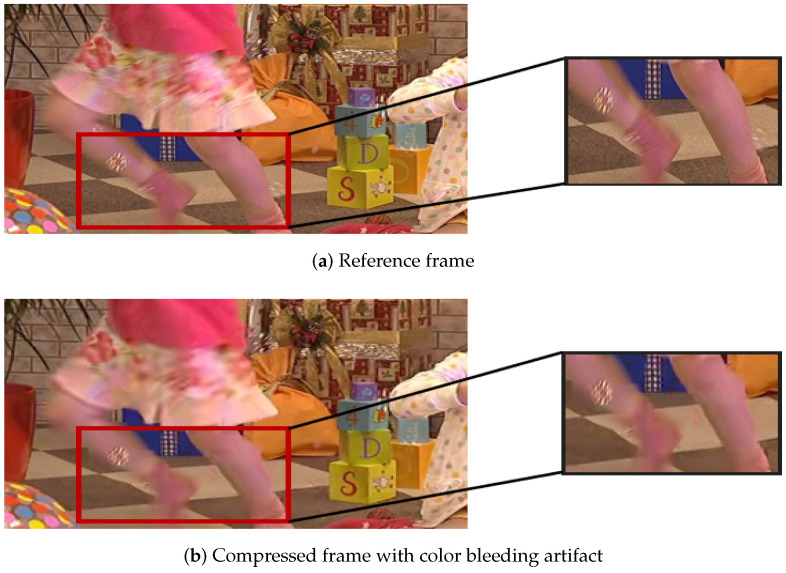
An example of color bleeding.

(1) Blurring: Modern video compression techniques involve a frequency transformation step followed by a quantization process, which usually eliminates small amplitude transformation coefficients. Because the energy of natural visual signals is concentrated at low frequencies, quantification reduces high frequency energy in such signals. It results in a significant blurring artifact in the reconstructed signal. Besides, de-blocking filter also leads to blurring artifact. Visually, blurring typically manifests itself as damage to spatial structure or reduced sharpness at edge or texture areas in images [22]. A blurring artifact example is shown in Figure 2b, which exhibits the spatial loss of the building field.

(2) Blocking: Standard video compression techniques utilize blocks of different sizes as basic units for frequency transformation and quantization. The quantization errors introduced in each block are presented in different forms, which result in discontinuities on block boundaries. In decoded videos, different forms of the blocking artifact are demonstrated, such as mosaic artifact, ladder artifact and pseudo-edge artifact. Blocking refers to discontinuities on the boundaries of adjacent blocks. The visual shape of blocking depends on the region where blocking occurs [26]. A blocking artifact example is shown in Figure 3b, which demonstrates the visual blocks of the face field.

(3) Ringing: When the quantization error of the high-frequency component corresponding to strong edges of image occurs, a corrugated pseudo-boundary will appear near the strong edge. With high-contrast edges, the ringing artifact is the most obvious in areas with smoother textures during the reconstruction process. Ringing shows ripple or vibration structures near the strong edge [20]. A ringing artifact example is shown in Figure 4b, in which the marked letters show the phenomenon of boundary ripples.

(4) Color bleeding: Color bleeding is the result of coarse quantification of chroma information. Color diffusion occurs in areas with very large chroma variation, resulting in blurring of chroma. After compression, due to low resolution of the color channel, interpolation operation is inevitably involved in the rendering process, which results in additional inconsistent color diffusion. Color bleeding is the result of inconsistent image rendering between brightness and chromaticity channels. A color bleeding artifact example is shown in Figure 5b, which exhibits a color diffusion in the marked rectangular field.

#### 2.1.2. Correlation between PEAs and Visual Quality

**Figure 6:**
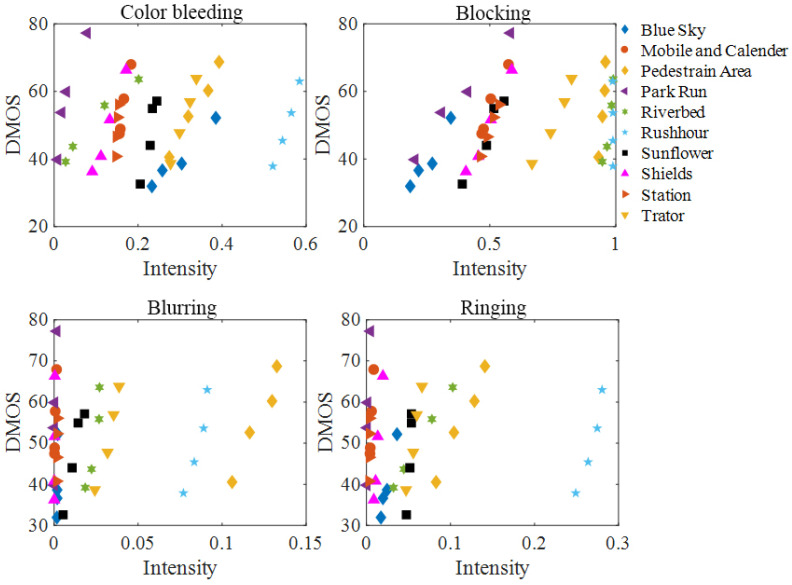
The scatterplot of each PEA intensity and DMOS on the LIVE Video Quality Database.

To verify the effects of four typical types of PEAs on visual quality, the correlation between PEAs and visual quality is studied. The sensitivity of human eyes to different types of PEAs is different. In [28], the blocking and blurring artifacts were observed to show significant impacts on visual quality of compressed videos. To explore the correlation between PEAs and compressed video quality [29], it is necessary to adopt a PEA detection algorithm. In this work, a PEA recognition model [1] is adopted, which can detect different types of PEAs with superior performance. The intensities of color bleeding, blocking, blurring and ringing are measured on the LIVE Video Quality Database [30], which consists of 10 reference videos and 40 compressed videos. The scatterplot of each PEA intensity value and video subjective quality score DMOS is shown in Figure 6, where the abscissa is PEA intensity, the ordinate represents DMOS value and each legend denotes compressed videos.

In addition, to study the influence of PEAs on video quality, it is necessary to analyze compressed videos with different distortion degrees. As can be seen from Figure 6, for different compressed videos with same content, there is a positive correlation between four PEA intensity values and their subjective quality scores, respectively, that is, the higher the PEA intensity value, the higher the DMOS value, the worse the video quality will be. Therefore, the existence of PEAs will reduce the quality of compressed videos. In order to further study the overall correlation between four types of PEAs and compressed video quality, the Pearson Linear Correlation Coefficient (PLCC) and Spearman Rank Correlation Coefficient (SRCC) of four PEA intensity values and their DMOS values are listed in Table 1, where the optimal and suboptimal correlation are represented in bold.

The above results show that the PLCC and SRCC of blocking artifact are the best, and that of blurring artifact are second best. Therefore, the blocking and blurring artifacts are the most important factors leading to the deterioration of compressed video quality. It also confirms the finding that the human eye shows the highest sensitivity to the blocking and blurring artifacts.

### 2.2. Video Saliency Detection with ACLNet

Visual saliency is an inherent attribute of HVS and is also a key factor affecting video perceptual quality [31]. The advantages of introducing visual saliency into video quality assessment are primarily reflected in two aspects: first, it allocates constrained hardware resources to more significant regions, and second, video quality analyze considering visual saliency is more consistent with human visual perception. Therefore, we select ACLNet as our video saliency model based on comprehensive comparison and analysis of popular video saliency models. ACLNet has strong applicability and its high real-time processing speed.

ACLNet combines attention module to improve features extracted by CNN [32], and utilizes a convLSTM [33] to obtain temporal characteristic. Then, convLSTM [33] is employed to model the temporal characteristic of this sequential issue, which is completed by merging memory units with gated operations. Finally, saliency maps of all frames are summarized as video saliency map. In addition, ACLNet uses the first five convolution blocks of VGG16 [34] and removes pool4 and pool5 layers to preserve more spatial details [22]. Saliency map can be expressed as

(1) $$\begin{array}{c} S_{i}=f_{s}\left(F_{i}\right), \end{array}$$

where $S_{i}$ refers to saliency map of the *i*-th frame. $f_{s}\left(\cdot \right)$ represents saliency algorithm.

### 2.3. Image Patch Segmentation

**Figure 7:**
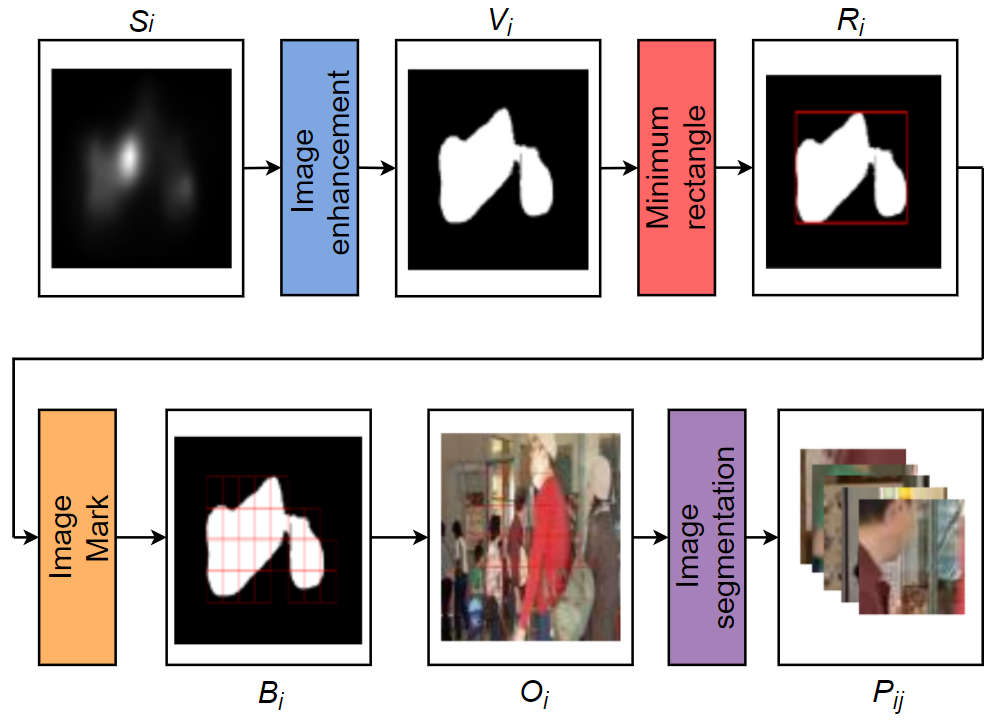
The process of image patch segmentation.

To make ease of PEA detection, saliency regions are segmented into image patches, which is shown in Figure 7. First, the saliency map of each frame is binarized. In this work, we adopt grayscale transformation to obtain a appropriate threshold. The saliency regions below the threshold are ignored. All bright regions in enhanced map are clipped into patches. We utilize 72 × 72 as patch size, in accordance with the input patch size of our PEA detection module. To minimize the number of image patches, the connected regions in the binary image are framed by smallest circumscribed rectangles. $R_{i}$ represents the binary images with minimum rectangle. The binary image marked with 72 × 72 square is denoted as $B_{i}$. All clipped image patches are grouped for PEA detection. The relationship between the original image marked with 72 × 72 square and image patches is calculated as follows:

(2) $$\begin{array}{c} \left\{O_{i_{j}},j\in \left[1...N\right]\right\}=P_{\mathrm{ij}}, \end{array}$$

where $O_{i}$ represents the original images marked with 72 × 72 square. $O_{i_{j}}$ denotes the *j*-th original image marked with 72 × 72 square, respectively. *N* (j∈[1…N]) is the total number of original images marked with 72 × 72 square. $P_{\mathrm{ij}}$ refers to all the clipped image patches of the video and is grouped for PEA detection.

### 2.4. PEA Detection

**Figure 8:**
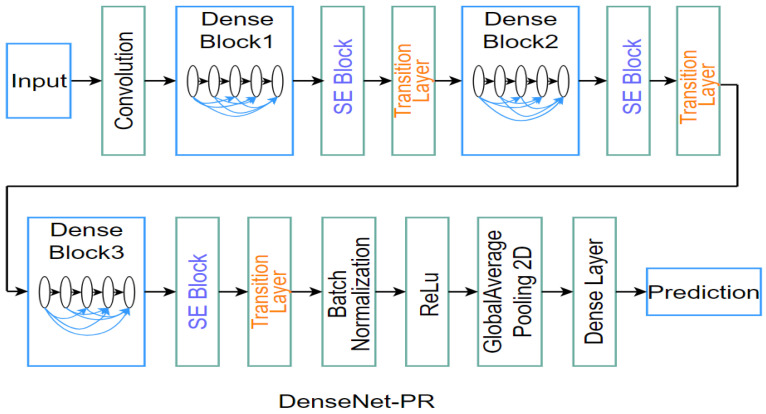
The structure of the DenseNet-PR [1]. (© 2020 IEEE)

To detect four typical types of PEAs, we utilize our DenseNet for PEA Recognition (Dense Net-PR) [1]. The specific structure of the DenseNet-PR is shown in Figure 8. First, Dense Block contains many layers, where the size of feature maps of each layer is the same. A feedforward fashion is utilized to establish connections between layers. The input of each layer is the feature maps of all previous layers, and the output feature maps of each layer are delivered to all subsequent layers. The nonlinear transformation function between layers is composed of Batch Normalization (BN), a rectified linear unit and a 3 × 3 convolution. Low computational complexity of the algorithm can be achieved by inserting a 1 × 1 convolution as the bottleneck layer before 3 × 3 convolution. The essence of this operation is to decrease the number of input feature maps. Additionally, we integrated the 3 × 3 convolution into a 3 × 3 and a 1 × 1 pointwise convolution to learn deeper features of feature channel. Second, we inserted a Squeeze and Excitation (SE) Block between each Dense Block and the transition layer to highlight vital characteristics of training set. Because this process also reused critical features of the transition layer, recognition accuracy is improved. Third, the transition layers are composed of a BN layer, a 1 × 1 convolutional layer and a 2 × 2 average pooling layer, where the 1 × 1 convolutional layer can decrease the number of feature maps. Finally, we utilize softmax classifier to return a list of probabilities. The label with the largest probability is chosen as the final classification.

The DenseNet-PR alleviates the vanishing-gradient problem, enhances feature propagation and greatly reduces the number of parameters. Based on the DenseNet-PR architecture, we randomly choose 50,000 ground-truth PEA samples to individually train four types of PEA recognition models from our subject-labeled database, which is composed of 324 compressed videos containing various PEAs [1]. The ratio of training sets and testing sets is 75:25 in these samples. Stochastic Gradient Descent (SGD) is adopted and the batch size is 256. The momentum is set to 0.9000. 0.0001 is the value of weight decay. The learning rate is adjusted following the schedule in [35] and its initial value is 0.1. The weight is initialized according to [35]. The depth and width of the DenseNet-PR network are set to 46 and 10, respectively. Based on the DenseNet-PR network, we individually trained four types of PEA recognition models to detect the presence of PEAs in image patches. It is worth mentioning that multi-objective classification is not utilized here because different types of PEAs may be coexist in one patch. Finally, based on the above models, a list of probabilities of each 72 × 72 patch are obtained to measure the PEA intensities, namely, $I_{\mathrm{ij}}$. Then, we can calculate the intensity of each PEA for a video sequence. We calculate the PEA intensity value of each patch and assume that the intensity of each pixel in the patch is equal to the intensity of the patch. For a few pixels that belong to overlapping patches, we use their average intensity values as the intensity values of these pixels. Finally, the intensity of each PEA of each video is calculated as follows:

(3) $$\begin{array}{c} I_{{\mathrm{frame}}_{k}}=\frac{1}{N_{\mathrm{pixel}}}\sum_{n=1}^{N_{\mathrm{pixel}}}I_{\mathrm{ij}}, \end{array}$$

(4) $$\begin{array}{c} I_{V_{k}}=\frac{1}{N_{\mathrm{frame}}}\sum_{n=1}^{N_{\mathrm{frame}}}I_{{\mathrm{frame}}_{k}}, \end{array}$$

where $N_{\mathrm{pixel}}$ refers to the total number of pixels in the saliency region of each frame. $I_{{\mathrm{frame}}_{k}}$ denotes the intensity value of the *k*-th type of PEAs per frame. $N_{\mathrm{frame}}$ is the total number of video frames. $I_{V_{k}}$ represents the intensity values of the *k*-th type of PEAs of each video, respectively.

### 2.5. Video Quality Prediction

**Figure 9:**
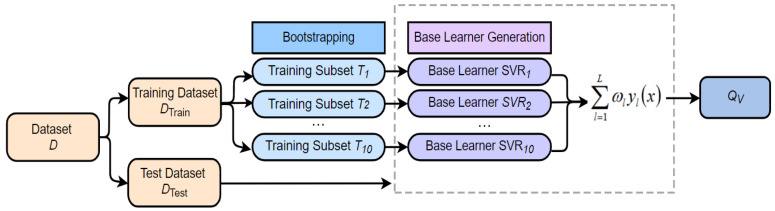
SVR model based on bagging.

To improve the generalization ability of our proposed SAAM metric, we design an ensemble model using Support Vector Regression (SVR) model based on Boostrap Aggregating (Bagging) as shown in Figure 9.

After obtaining the intensity values of PEAs, ensemble learning model is adopted to map the intensity values of four types of artifacts to MOS|DMOS values. First, for anyone selected VQA database, we form a complete data set *D* by matching the intensity values of four types of artifacts to MOS|DMOS, which can be expressed as

(5) $$\begin{array}{c} D=\{\left(I_{V_{1}},{\mathrm{MOS}}_{1}|{\mathrm{DMOS}}_{1}\right),\left(I_{V_{2}},{\mathrm{MOS}}_{2}|{\mathrm{DMOS}}_{2}\right),\ldots ,\left(I_{V_{k}},{\mathrm{MOS}}_{m}|{\mathrm{DMOS}}_{m}\right)\}, \end{array}$$

where ${\mathrm{MOS}}_{m}|{\mathrm{DMOS}}_{m}$ represents the quality score MOS|DMOS of the *m*-th compressed video. Second, the date set is randomly split into training set $D_{\mathrm{Train}}$ and testing set $D_{\mathrm{Test}}$ at a ratio of 80:20, and 10 sub-training sets [$T_{1}$, $T_{2}$, ..., $T_{10}$] are resampled from the $D_{\mathrm{Train}}$. Note that the sub-training set [$T_{1}$, $T_{2}$, ..., $T_{10}$] and $D_{\mathrm{Train}}$ contain the same number of samples. Then, we train 10 SVR models as the base learners through [$T_{1}$, $T_{2}$, ..., $T_{10}$], that is, the intensity values of four types of PEAs for video sequences are fed into SVR, and then SVR output a predicted value for each video. In this work, we chose the radial basis function as the kernel of SVR due to its better performance. Next, $D_{\mathrm{Test}}$ is utilized to evaluate the performance of these base learners by the PLCC between the predicted quality scores and the true quality scores. Finally, the final prediction result $Q_{V}$ is obtained as follows:

(6) $$\begin{array}{c} Q_{V}=f\left(\sum \sum I_{\mathrm{ij}}\right)=\sum_{l=1}^{L}\omega_{l}y_{l}\left(x\right), \end{array}$$

(7) $$\begin{array}{c} \sum_{l=1}^{L}\omega_{l}=1\;\omega_{l}\geq 0, \end{array}$$

where f(·) refers to summation operation. *x* represents $D_{\mathrm{Test}}$. $y_{l}\left(x\right)$ refers to the prediction output of the *l*-th base learner. *L* is the number of base learners, which value is 10. $\omega_{l}$ denotes the weight of the *l*-th base learner. In this work, we set the weights of based learners with the top three PLCC to 1/3, and the weights of the remaining base learners are set to 0.

## 3. Experiments and Discussions

To evaluate the performance of our proposed algorithm, it is examined on four publicly and widely used Video Quality Databases (VQD): LIVE, CSIQ, IVP and FERIT-RTRK. Among them, the compressed videos generated by H.264 encoder are utilized here to evaluate the performance of SAAM. The LIVE VQD contains 40 compressed videos with a resolution of 768 × 432 [30]. The CSIQ VQD contains 36 compressed videos with a resolution of 832 × 480 [36]. The IVP VQD contains 40 compressed videos with a resolution of 1920 × 1088 [37]. The FERIT-RTRK VQD consists 30 compressed videos with a resolution of 1920 × 1080 [38]. Based on the compressed videos from the four VQA databases, we individually form four complete data sets by matching the intensity values of four types of artifacts to their MOS|DMOS as described in Section 2.5.

To show the superiority of our method, it is compared with typical video quality metrics including PSNR, SSIM [3], MS-SSIM [39], STRRED [4], SpEED-QA [5], BRISQUE [40], NIQE [41] and VIIDEO [11]. Among them, PSNR, SSIM and MS-SSIM are FR metrics. STRRED and SpEED-QA are RR metrics, and BRISQUE, NIQE and VIIDEO are NR metrics. All methods are compared in terms of the PLCC and SRCC, which characterize the correlation between VQA results and MOS|DMOS values. The results are reported in Table 2 and Table 3. Among them, as our SAAM metric is based on machine learning, to fairly verify its performance, the result of our metric is the median value of 15 repeated processes. In addition, the overall performance of each VQA algorithm on the four video databases is listed in the last column of the tables, expressed by weighted PLCC and SRCC. The weight of each database depends on the number of distorted videos in the database, and the optimal performance is given in bold.

From the tables, our algorithm delivers strong competitive performance on these datasets. First, the PLCC of the proposed SAAM approach outperforms all of compared methods on the four databases. Second, on the CSIQ database, the SRCC of the SAAM outperforms that of PSNR, SSIM, BRISQUE, NIQE and VIIDEO and is competitive with the performance of MS-SSIM, STRRED and SpEED-QA. Finally, the overall performance of the SAAM is better than all of compared VQA methods. The experimental results also show that there is a strong correlation between the PEA intensity and subjective quality of a compressed video, and the PEAs affect the viewing experience of end users.

To further verify the generalization of the proposed algorithm, we also studied cross-dataset evaluation in Table 4. It can be observed that when CSIQ VQD is used as the testing set, the performance of using LIVE VQD as the training set is relatively better than that of utilizing FERIT-RTRK VQD as the training set. The most likely reason may be that the resolutions of LIVE and CSIQ databases are very close, but the difference of resolutions between FERIT-RTRK and CSIQ VQD is relatively bigger. In addition, the subjective quality scores provided by LIVE and CSIQ VQD are MOS values, while the scores provided by FERIT-RTRK VQD are DMOS values. Therefore, different scoring standards of the subjective quality scores may also cause differences in cross-database performance. Generally, the performances of image processing algorithms are usually not very good in cross-database performance verification, especially considering that the video resolutions and contents of various databases are different. At this point, our performance of cross-database experiment is acceptable. It also shows that our proposed model has good generalization and robustness. In our algorithm, we can adjust the PEA recognition models to further improve the recognition accuracy of artifacts and increase the correlation between SAAM results and MOS|DMOS, which will become our next work.

Besides, we also perform ablation experiments on CSIQ VQD to verify the advantages of saliency detection. We crop the whole frame into patches for PEA detection, and the size of data is 1.11 GB. We only retain the patches containing saliency regions for PEA detection, the size of data is reduced to 0.37 GB and the storage is saved by 66.49%. The time consumption of PEA detection is reduced from 6.47 h to 1.50 h, saving by 76.82%, as shown in Table 5.

## 4. Conclusions

In this paper, we propose a NR-VQA metric called SAAM, based on the intensity values of four typical types of artifacts (i.e., blurring, blocking, ringing and color bleeding). To the best of our knowledge, this is the first work combining video saliency with artifacts detection to predict the quality of compressed video. The experimental results demonstrate that the proposed algorithm delivers competitive performance with common video quality metrics in different datasets. As future work, we plan to design a NR-VQA algorithm based on natural video statistics, which can detect more types of video PEAs.

## Figures and Tables

**Table 1:** Correlation between the PEA intensity and DMOS on the LIVE Video Quality Database.

Correlation	Blocking	Blurring	Ringing	Color Bleeding
PLCC	**0.5551**	**0.1921**	0.1900	0.1902
SRCC	**0.4208**	**0.1848**	0.1340	0.1109

**Table 2:** Performance comparison in terms of PLCC.

Methods	LIVE	CSIQ	IVP	FERIT-RTRK	Overall
PSNR	0.5735	0.8220	0.7998	0.7756	0.7383
SSIM	0.6072	0.8454	0.8197	0.6870	0.7406
MS-SSIM	0.6855	0.8782	0.8282	0.8724	0.8105
STRRED	0.8392	0.8772	0.5947	0.8425	0.7823
SpEED-QA	0.7933	0.8554	0.6822	0.6978	0.7586
BRISQUE	0.2154	0.5526	0.2956	0.7653	0.4335
NIQE	0.3311	0.5350	0.3955	0.5817	0.4505
VIIDEO	0.6829	0.7211	0.4358	0.3933	0.5651
SAAM	**0.9023**	**0.9244**	**0.8717**	**0.9499**	**0.9091**

**Table 3:** Performance comparison in terms of SRCC.

Methods	LIVE	CSIQ	IVP	FERIT-RTRK	Overall
PSNR	0.4146	0.8028	0.8154	0.7685	0.6928
SSIM	0.5677	0.8440	0.8049	0.7236	0.7328
MS-SSIM	0.6773	0.9465	0.7917	0.8508	0.8107
STRRED	0.8358	**0.9770**	0.8595	0.8310	0.8761
SpEED-QA	0.7895	0.9639	**0.8812**	0.7945	0.8587
BRISQUE	0.2638	0.5655	0.1051	0.7574	0.3961
NIQE	0.1769	0.5012	0.2351	0.4855	0.3362
VIIDEO	0.6593	0.7153	0.1621	0.3177	0.4667
SAAM	**0.8691**	0.8810	0.8413	**0.9429**	**0.8796**

**Table 4:** Cross-database validation.

Training Set	Testing Set	PLCC	SRCC
LIVE	CSIQ	0.7107	0.7290
FERIT-RTRK	CSIQ	0.4437	0.5302

**Table 5:** Ablation experiments on the CSIQ VQD database.

	Data Size	Time of PEAs Detection	PLCC	SRCC
Without Saliency	1.11 GB	6.47 h	0.9557	0.8929
With Saliency	0.37 GB	0.50 h	0.9244	0.8810

## Data Availability

Data available on request from the authors.
